# Fascin-1 and its role as a serological marker in prostate cancer: a prospective case–control study

**DOI:** 10.2144/fsoa-2021-0051

**Published:** 2021-06-30

**Authors:** Octavian Sabin Tătaru, Orsolya Martha, Felice Crocetto, Biagio Barone, Septimiu Voidazan, Angela Borda, Anca Sin, Adina Hutanu, Andrada Loghin, Ileana Sin, Daniel Porav-Hodade, Calin Bogdan Chibelean, Liliana Vartolomei, Giuseppe Lucarelli, Matteo Ferro, Virgil Gheorghe Osan, Carlo Buonerba, Mihai Dorin Vartolomei

**Affiliations:** 1 I.O.S.U.D., George Emil Palade University of Medicine & Pharmacy, Science & Technology, Targu Mures, Romania; 2 Department of Urology, George Emil Palade University of Medicine & Pharmacy, Science & Technology, Targu Mures, Romania; 3 Department of Neurosciences, Reproductive Sciences & Odontostomatology, University of Naples Federico II, Naples, Italy; 4 Department of Epidemiology, George Emil Palade University of Medicine & Pharmacy, Science & Technology, Targu Mures, Romania; 5 Department of Histology, George Emil Palade University of Medicine & Pharmacy, Science & Technology, Targu Mures, Romania; 6 Department of Cell & Molecular Biology, George Emil Palade University of Medicine & Pharmacy, Science & Technology, Targu Mures, Romania; 7 C.C.M.A.F., George Emil Palade University of Medicine & Pharmacy, Science & Technology, Targu Mures, Romania; 8 Department of Emergency & Organ Transplantation-Urology, Andrology & Kidney Transplantation Unit, University of Bari, Bari, Italy; 9 Division of Urology, European Institute of Oncology (IEO)-IRCCS, Milan, Italy; 10 Department of Oncology & Hematology, Regional Reference Center for Rare Tumors, AOU Federico II of Naples, Naples, Italy

**Keywords:** biomarker, Fascin-1, fascin actin-bundling protein 1, prostate cancer, serum

## Abstract

**Aim:**

This study aims to investigate any modification of serological FSCN1 in prostate cancer patients compared with patients without neoplasia.

**Material & methods:**

Clinical data and blood specimens from patients with and without prostate cancer were obtained. A quantitative sandwich ELISA method was used to determine serological values of FSCN1.

**Results:**

Although serum values of FSCN1 were dissimilar in the two cohorts of patients (6.90 vs 7.33 ng/ml), the difference was not statistically significant (p = 0.20). Serum values of FSCN1 stratified for Gleason score groups were not significantly distinguishable (p = 0.65). A negative correlation (rho = -0.331; p = 0.009) was reported between FSCN1 and age.

**Conclusion:**

Further studies are required to evaluate a possible diagnostic role of FSCN1 in prostate cancer.

## Lay abstract

FSCN1 is a potential novel biomarker that we investigated in patients with prostate cancer and evaluated in serum through a quantitative assay. Although FSCN1 serum values were dissimilar between patients with and without prostate cancer (with lower values in the first group), data are currently inconclusive. A negative correlation between FSCN1 and age was instead reported. Further studies are required to investigate a possible diagnostic role of FSCN1.

Prostate cancer (PCa) is the third-most diagnosed neoplasia worldwide and the fifth major cause of cancer death in the male population [1]. In 2021, around 248,530 patients are likely to be diagnosed with PCa in the USA alone, with an estimated number of 34,130 deaths [2]. Prostate-specific antigen (PSA) screening, although it suffers limitations related to fluctuating values linked to different factors (as drugs, inflammation or lifestyle habits), effectively identifies well-differentiated tumors [3–5]. Less-differentiated PCa, which increases cancer-specific death rates, are often not identified by PSA only, as most of the patients have normal PSA values [6]. Several blood or serum-based biomarkers used for diagnosis of PCa, such as the US FDA approved tests (PSA, fPSA, Prostate Health Index) [7–10], or clinical laboratory improvement amendments (CLIA)-approved biomarkers (4Kscore) [11–14], and also test pending approval, such as STHLM3 and STHLM3MRI [15–19] have been developed in recent years. These panels could provide an early detection of PCa, leading to better oncological outcomes and decreasing PCa mortality [20]. However, to date, no blood biomarkers have been able to establish a diagnosis of PCa.

## FSCN1 as biomarker

Fascin-1 (FSCN1) is an actin-bundling protein in the cytoskeleton of epithelial cells, with a low or absent expression in the majority of normal adult epithelia while its upregulation has been observed in several types of cancers [21,22]. FSCN1 has been studied, in numerous research initiatives, as a possible diagnostic marker or therapeutic target in metastatic cancers [23,24]. This protein has indeed been shown to be highly expressed in different types of human carcinoma with an unfavorable prognosis.

## FSCN1 in different cancers

Elevated expression of FSCN1 in colorectal and gallbladder cancer is an independent negative survival outcome factor, which correlates with an increased risk for disease recurrence and poor prognosis [25–29]. Similarly, high expression of FSCN1 has been identified in patients with increased risk for recurrence in breast and non-small-cell lung cancer [30–33]. In bladder cancer, overexpression of FSCN1 increases cell migration and promotes metastasis, correlating with increased risk of progression and predicting invasiveness and recurrence [34–37]. Serological and blood determination of FSCN1 has been studied in head and neck cancers, differentiating between healthy and cancer patients [38].

## FSCN1 in prostate cancer

In PCa patients, FSCN1 expression was experimentally investigated by immunohistochemistry in benign, localized and hormone-refractory PCa and found to be highly expressed in hormone refractory PCa and localized PCa, reporting that epithelial expression of FSCN1 is not statistically significant associated with Gleason score, pathologic stage or surgical margins [39]. In addition, FSCN1 expression is correlated with surgical margin status in PCa and its upregulation by PCa-associated lncRNA transcript 1 (PCAT-1), mediated through miR-145-5p, is pivotal in PCa cells proliferation, migration and invasion [40,41]. Furthermore, the oncoprotein N-Myc, which causes castration resistance in PCa, promotes the malignant progression of PCa in *in vitro* models through overexpression of FSCN1 [42]. As reported in recent immunohistochemical analyses of FSCN1 expression in PCa tissue, no correlation was found with Gleason score, tumor stage and PSA values. However, the analysis of FSCN1 using immunohistochemistry in prostate carcinoma glands (several hundred prostate specimens of all Gleason risk scores) found that only 8% of the tumors had more than 10% FSCN1 positivity, and the stromal tumor cells, and stromal levels of FSCN1 were highly increased in high Gleason score [43,44]. We have proposed two objectives to study FSCN1 in PCa. One is FSCN1 as a circulating marker in PCa and the other to use *FSCN1* mRNA data from publicly available transcriptomics data analysis to support the previously inconclusive statements regarding FSCN1 expression based on immunohistochemical data. To our knowledge, our research is the first study that aims to investigate any modification of FSCN1 serum levels in PCa patients compared with control patients. Any correlation regarding age, PSA values, PSA density, prostate volume, Gleason score and serum levels of FSCN1 is also analyzed.

## Materials & methods

### Patient selection

Our study is a prospective case–control study conducted between 1 April 2016 and 31 March 2018 in the Urology Department of the County Clinical Hospital in Targu Mures, affiliated to George Emil Palade University of Medicine, Pharmacy, Science and Technology of Targu Mures, Romania. Blood specimens were collected according to a predetermined standard operating procedure before prostate biopsy [45]. Whole blood was allowed to clot before serum was separated by centrifugation. Serum aliquots were stored at -80°C until samples were processed, according to Semjonow *et al.* [46]. Among the patients involved, 76 met the eligibility criteria for this study: no prior prostate surgery and biopsy, no bacterial acute or chronic prostatitis, no use of 5-α reductase inhibitors, availability of serum samples and corresponding clinical data and completion of at least a 12-core template biopsy after enrollment. The final study cohort included 62 consecutive male patients with ages between 55 and 75 years old with clinical and pathological data proven PCa. Prostate biopsy was performed following an abnormal digital rectal examination and elevated PSA levels (>4 ng/ml), according to the Vienna Nomogram [47]. Sixty-one aged-matched (within 3 years) control male patients, were enrolled as control group, with no abnormal digital rectal examination, PSA: 0–4 ng/ml, no other previous prostate surgery and no other known malignant disease and patients with elevated PSA (over 4 ng/ml) and negative prostate biopsy. Patients with PCa at biopsy underwent treatment and were included in a regular follow-up procedure according to the European guideline recommendations [48].

### ELISA serum determination

In order to evaluate FSCN1 serum levels, a quantitative sandwich ELISA method was used, following the manufacturer’s instructions (US Biological, Human Fascin, MA, USA), with a detection range of 0.312–20 ng/ml. After blood collection, the tubes were allowed to clot and centrifuged at 3500 rpm Serum was aliquoted and stored at -80°C, and successively analyzed on automated DSX Dynex ELISA System (Dynex Technology, VA, USA) [49].

### Statistics

Statistical analysis was performed with Graph-pad version 3.6 (CA, USA). Evaluation of the normality of continuous variables was assessed with the Kolmogorov–Smirnov test. For continuous variables (expressed as mean ± SD), we used the Student *t*-test to assess the differences between means, whereas for differences between variables expressed by median and range, we applied nonparametric tests: the Mann–Whitney test and Kruskal–Wallis test. Dunn’s multiple comparison tests were used to find the statistically significant differences between groups. Spearman Rank Correlation (Spearman’s rho) test was used to identify a relationship between age and FSCN1 values [50]. The threshold for statistically significant result was set for p = 0.05.

### *FSCN1* mRNA data from publicly available transcriptomics data analysis

CANCERTOOL (http://web.bioinformatics.cicbiogune.es/CANCERTOOL/index.html) was explored for publicly available transcriptomics data analysis [51]. The GEPIA2 database (http://http://gepia2.cancer-pku.cn) was used to evaluate the expression of *FSCN1* gene in a large cohort of PCa patients [52]. In this web-based resource, the data from the Cancer Genome Atlas (TGCA) and Genotype-Tissue Expression are available for validation analysis. The prostate adenocarcinoma (PRAD) cohort includes 492 PRAD and 152 normal prostate specimens. PRAD-TGCA subgroup analyses (Gleason score and nodal metastasis status) were performed using the UALCAN portal (http://ualcan.path.uab.edu/index.html) [53].

## Results

**Figure 1.**
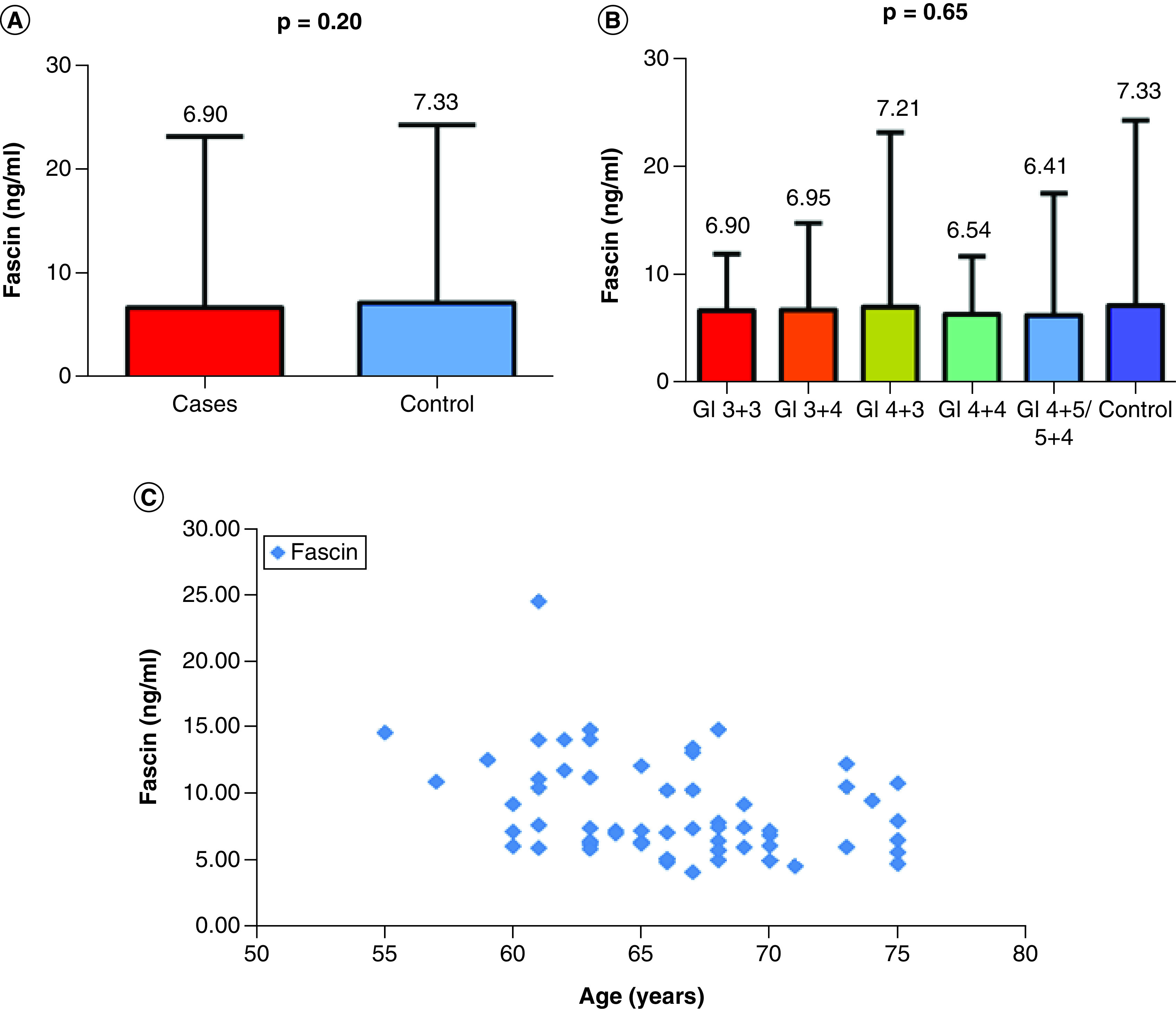
FSCN1 serum values in study population. **(A)** The serum FSCN1 levels in prostate cancer patients (cases) versus controls by applying Mann–Whitney test in order to compare the median FSCN1 serum levels between the two groups. **(B)** The levels of FSCN1 for Gleason score groups in PCa patients when compared with controls performing Kruskal–Wallis test to determine the statistically significant difference between the medians of Gleason score groups. **(C)** The negative correlation (Spearman test) between FSCN1 and age for the control group (rho = -0.331; p = 0.009).

Clinical and biochemical characteristics of patients and controls are shown in Table 1. Age did not differ between the two studied groups (mean: 67.74 and 66.13 years; p = 0.06). Similarly, no differences were found for prostate volumes among PCa patients and controls (median of 37cc for PCa patients and 33cc for controls; p = 0.18). As expected, statistically significant difference was reported for PSA (median of 18.83 vs 1.82 ng/ml; p < 0.0001) and PSA density (median of 0.52 vs 0.06 ng/ml^2^; p < 0.0001). No statistically significant difference (p = 0.20) was reported in FSCN1 serum values among PCa patients (median value of 690 ng/ml) and controls (median value of 7.33 ng/ml) (Figure 1A). Analogously, serum values of FSCN1 stratified for Gleason groups and compared with controls reported no statistical significance as well (p = 0.65), with a median of 6.90 ng/ml for Gleason 3+3; 6.95 ng/ml for Gleason 4+3; 7.21 ng/ml for Gleason 4+3; 6.54 ng/ml for Gleason 4+4; and finally, a median of 6.41 ng/ml for Gleason 4+5 or 5+4 (Figure 1B). Higher values of serum FSCN1 were reported in younger patients, with progressively decreasing values for older patients, reporting a statistically significant negative correlation between FSCN1 and age (rho = -0.331; p = 0.009) (Figure 1C).

**Table 1.** Comparison of control versus PCa patient’s characteristics.

Characteristic	Control	PCa	p-value
Age (years) Mean (SD), (minimum–maximum)	66.13 (4.81) (55–75)	67.74 (4.81) (55–75)	0.06^†^
PSA Median (minimum–maximum)	1.82 (0.36–22.90)	18.83 (0.04–6420)	0.0001^‡^
PSA density Median (minimum–maximum)	0.06 (0.01–0.63)	0.52 (0.0–0.01)	0.0001^‡^
Prostate volume (cc^3^) Median (minimum–maximum)	33 (10–120)	37 (16.80–103.4)	0.18^‡^
DRE	Negative	Positive	

† Student test.

‡ Mann–Whitney test.

DRE: Digital rectal examination; PSA: Prostate-specific antigen; SD: Standard deviation.

**Figure 2.**
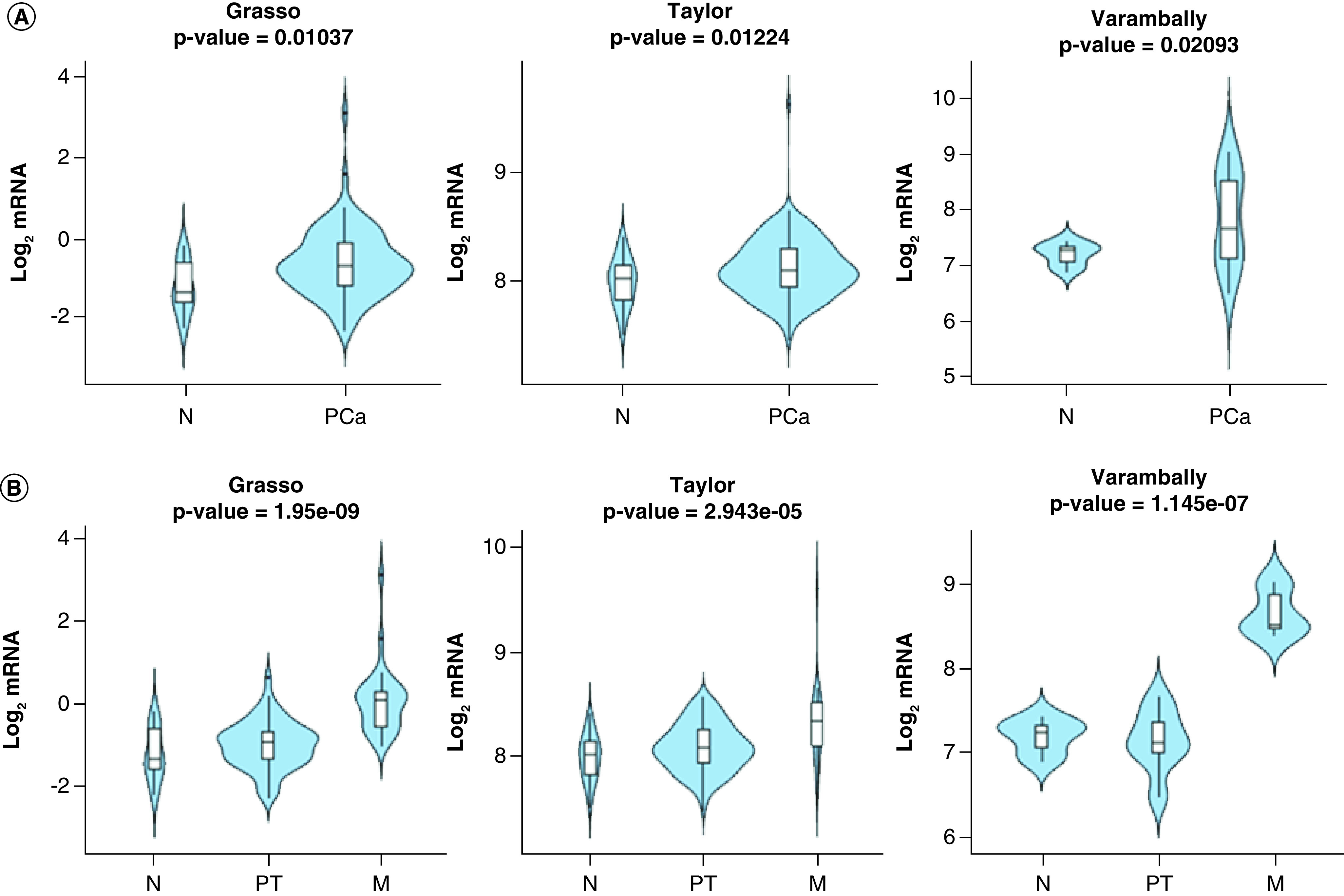
Violin plots depicting FSCN1 gene expression in different datasets. **(A)** Between nontumoral (N) and prostate cancer (PCa) specimens in the indicated datasets. The Y-axis represents the Log2-normalized gene expression (fluorescence intensity values for microarray data or, sequencing reads values obtained after gene quantification with RNA-Seq by expectation maximization (RSEM) and normalization using upper quartile in case of RNAseq). A Student *t*-test is performed in order to compare the mean gene expression between two groups. **(B)** Among N, primary tumor and metastatic PCa specimens in the indicated datasets. The Y-axis represents the Log2-normalized gene expression (fluorescence intensity values for microarray data or, sequencing reads values obtained after gene quantification with RSEM and normalization using upper quartile in case of RNAseq). An ANOVA test is performed in order to compare the mean gene expression among two groups. M: Metastatic; N: Nontumoral; PT: Primary tumor.

**Figure 3.**
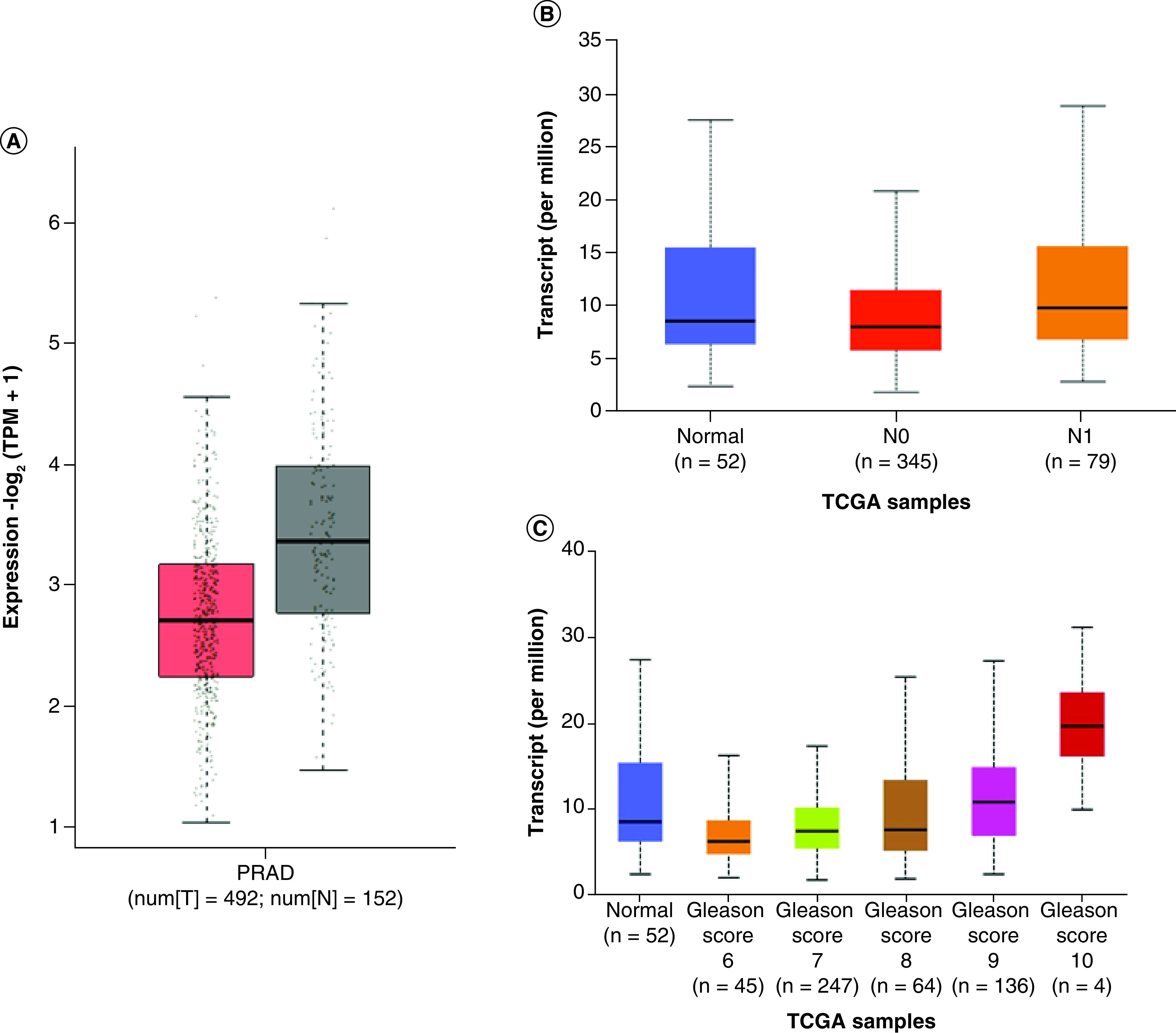
*Box plots depicting FSCN1* gene expression in TGCA-prostate adenocarcinoma dataset. **(A)** Expression between nontumoral and prostate cancer specimens in the TGCA-prostate adenocarcinoma (PRAD) dataset. **(B)** In the TGCA-PRAD dataset, based on nodal metastasis status. **(C)** In the TGCA-PRAD dataset, based on Gleason score. N: Nontumoral; PRAD: Prostate adenocarcinoma; T: Prostate cancer tumor.

Data mining of Grasso, Taylor and Varambally datasets showed overexpression of *FSCN1* mRNA in PCa tissue compared with normal tissue (Figure 2A), particularly in metastatic samples (Figure 2B) [54–56]. We evaluated these findings in the TGCA-PRAD dataset and we found no statistically significant difference between the two groups (p > 0.05), although *FSCN1* was overexpressed in tumors with nodal metastases and with high Gleason scores (Figure 3A–C).

## Discussion

Our results showed that serum levels of FSCN1 did not differ between PCa patients and controls, and these findings are in accordance with the results of data mining performed on TCGA-PRAD dataset. In contrast, similar serum FSCN1 determinations, performed in patients diagnosed with lung cancer, laryngeal carcinoma and hepatocellular carcinoma, were correlated to tumor aggressiveness [32,38,49]. Teng *et al.* were the first to investigate serum FSCN1 in patients with cancer, utilizing an ELISA kit for human FSCN1 from USCN Life Science (TX, USA) and reporting higher values in patients with NSCLC compared with healthy controls (which reported a median value of 3 ng/ml) [33]. Contrary to our study, serum samples were diluted a 100-fold, while we performed a 5–1 dilution, obtaining FSCN1 serum values that were in the lowest part of kit detection range. In order to avoid undetected FSCN1 in serum due to hyper dilution, we did not perform further dilution. As results, our mean FSCN1 serum level reported for healthy controls was 7.33 ng/ml. However, normal FSCN1 serum levels were shown to be 9.43 ng/ml, in a recently published article [49]. Similarly, FSCN1 serum levels identified by Elewa *et al.* in healthy males was 9.9 ng/ml while patients with hepatocellular carcinoma showed a mean FSCN1 serum level of 14.8 ng/ml which was significantly higher compared with levels reported in controls and in cirrhotic participants [49,57]. However, also in those cases, methodologies were different as FSCN1 serum levels were determined utilizing different ELISA kits. Other potential biases could lie in the higher mean age of controls and higher number of included determinations. Among previous studies reported, a level of FSCN1 >15 ng/mg could represent a potential clinically significant cutoff; however, our results differ and our FSCN1 serum levels are lower than those already published. Moreover, it was recently demonstrated that targeted inhibition of FSCN1 could interfere with tumor invasion and metastatic potential [58,59]. The analysis of FSCN1 using immunohistochemistry in prostate carcinoma glands found that only 8% of PCa had more than 10% FSCN1 positivity; FSCN1 expression, however, did not correlate with Gleason score, tumor stage, serum PSA levels or biochemical relapse following surgery [43]. The analysis of *FSCN1* mRNA expression in different PCa datasets showed a quite limited diagnostic role of FSCN1, although increased in metastatic disease [60]. These data could, however, be useful in order to deliver a proper and prompt therapy in this subset of patients [61,62]. Interestingly, the analysis of Kaplan–Meier curves in TCGA-PRAD datasets showed that the disease-free survival was significantly decreased in patients with high-tissue levels of *FSCN1* transcripts. We do not report higher serum levels of FSCN1 in patients with high Gleason score when compared with low or intermediate Gleason score. Consequently, contradictory evidence is reported in the literature regarding tumor expression of FSCN1, prompting a more detailed analysis. The relationship between age and serum levels of FSCN1 was contradictory as well with FSCN1 expression in neoplastic tissue of lung cancer that reported statistically significant differences among patients’ age groups (≤61 vs >61 years; p = 0.032), while no significant differences were reported in age groups for NSCLC patients (p > 0.05) [63,64]. Similarly, for patients with spinal or intracranial meningioma, it has been demonstrated that no relation exists between the tissue expression of FSCN1 and age (<60 vs ≥60 years; p = 0.693) [65]. Evenly, in patients with cholangiocarcinoma or with bladder cancer, there was no statistically significant difference between FSCN1 expression and age (p > 0.05) [66,67]. Conversely, in patients with esophageal squamous cell carcinoma, FSCN1 correlated with age groups (<50 vs ≥50 years; a p < 0.05) [68]. In our study, we reported a negative correlation between age and serum levels of FSCN1, meaning that in younger male control patients, we may find higher levels of FSCN1 and *vice versa*. In the follow-up regimen, the serum levels of FSCN1 were not measured after radical prostatectomy or other therapeutic interventions. The data available at this point are scarce and, as previously reported, with opposite results coming from different research studies, both tissue analysis and blood analysis, meaning that a definitive conclusion cannot be drawn.

A number of limitations have to be addressed for this study. The potential clinical relevance of a circulating biomarker is to follow its serum levels after a therapeutic intervention. Having in mind the above disappointing results, PCa patients were not followed up after treatment. Undoubtedly, a larger cohort of patients could increase the statistical strength of the study, nevertheless, we included 61 controls and 62 patients with PCa, confirming the already known data about serum levels of FSCN1 in healthy patients. The FSCN1 results were obtained using two kits (from the same producer), the samples were not measured as duplicates and the need for two kits derived from the fact that the samples were obtained during 2 years. In controls, PCa was excluded based on clinical examination and PSA, and several patients have had negative prostate biopsy performed due to elevated PSA. Regarding PCa, apart from already published data that demonstrated overexpression in tumor tissue using immunohistochemistry, our results on FSCN1 as a new biomarker in PCa patients must be seen with limited potential.

## Conclusion

Here, serum FSCN1 levels in PCa patients, compared with controls, had no statistical significance. There is a negative correlation between FSCN1 and age limited to the control group, leading us to conclude that FSCN1 serum levels differ according to age. All outcomes studied point to the fact that a possible role of FSCN1 as a reliable marker for the diagnosis of PCa has unsubstantiated results. It is more likely that more trials, involving patients and controls, might establish a possible role of serum FSCN1 in evaluating the outcome, survival, diagnosis and prognosis of metastasis in PCa patients.

## Future perspective

The role of FSCN1 in PCa is still controversial. Nevertheless, due to the prognostic and therapeutic role of this actin-bundling protein in several cancers, it is possible that the absence of correlation between FSCN1 and PCa could be merely linked to a temporary lack of knowledge in this field. FSCN1 could represent, therefore, alone or in combination, a promising biomarker in the next future in the diagnosis and the prognosis of PCa. Further studies are required to explore the diagnostic/prognostic possibilities related to the expression of this protein.

**Summary points**

- Fascin-1 (FSCN1) is an actin-bundling protein in the cytoskeleton of epithelial cells, upregulated in several types of cancers.
- In PCa FSCN1 seems to be highly expressed in hormone refractory PCa and localized PCa, with, in addition, an expression correlated to surgical margin status.
- Immunohistochemistry in prostate carcinoma glands found that only 8% of tumors had more than 10% of FSCN1 positivity.
- We performed a prospective case–control study on 62 PCa patients and 61 age-matched control subjects, confronting FSCN1 serum values.
- Although serum values of FSCN1 were dissimilar in the two cohorts of patients (6.90 vs 7.33 ng/ml), difference was not statistically significant.
- Serum values of FSCN1 stratified for Gleason score groups were not significantly distinguishable.
- A statistically significant negative correlation (rho = -0.331; p = 0.009) between FSCN1 expression and age was reported.
- Further studies are required to evaluate a possible diagnostic role of FSCN1 in PCa.
